# Validating At-Home Urinary Hormone Measurements in Postpartum and Perimenopause Fertility Transitions

**DOI:** 10.1089/whr.2024.0157

**Published:** 2025-03-28

**Authors:** Thomas P. Bouchard, Patricia K. Doyle-Baker, Paul J. Yong, Richard Fehring, Mary Schneider

**Affiliations:** ^1^ Department of Family Medicine, University of Calgary, Calgary, Canada.; ^2^ Department of Obstetrics and Gynecology, University of British Columbia, Vancouver, Canada.; ^3^ Faculty of Kinesiology, University of Calgary, Calgary, Canada.; ^4^ College of Nursing, Marquette University, Milwaukee, Wisconsin, USA.

**Keywords:** ovulation, menstrual cycle, perimenopause, postpartum, estrone-3-glucuronide (E_1_3G), luteinizing hormone (LH)

## Abstract

**Background:**

Measuring quantitative menstrual cycle hormones at home may help women better understand their postpartum and perimenopause fertility transitions, but these quantitative fertility monitors require validation.

**Materials and Methods:**

This study included 16 North American women, aged 28–51, during either the postpartum (*n* = 8, cycles = 18) or perimenopause (*n* = 8, cycles = 35) fertility transitions testing daily first-morning urine testing with both the Mira Monitor and ClearBlue Fertility Monitor (CBFM) along with menstrual cycle parameter tracking. The main outcome measures were a rise in estrone-3-glucuronide (E_1_3G) and luteinizing hormone (LH) urine hormone values from the Mira monitor correlated to low, high, or peak values on the CBFM.

**Results:**

Both in the postpartum and perimenopause transitions, the identification of the day of ovulation based on the LH surge on the Mira and CBFM monitors was highly correlated (R = 0.94 and 0.83, *p* < 0.001). The E_1_3G levels on the Mira monitor were significantly higher for a CBFM reading of “High” compared with “Low” for both the postpartum and perimenopausal cycles (all *p* < 0.001). Similarly, the LH levels on the Mira monitor were significantly higher for a CBFM reading of “Peak” (LH surge) compared with “High” for both the postpartum and perimenopausal cycles (all *p* < 0.001).

**Conclusions:**

The LH surge and levels of E_1_3G in urine identified on the quantitative Mira fertility monitor strongly correlate to the LH surge and the shift from low to high on the CBFM during the postpartum and perimenopause transitions.

## Introduction

In the past two decades, there has been a significant evolution in new technologies for at-home personalized fertility monitoring.^1^ The industry standards for ovulation prediction with urinary hormones have included the ClearBlue Fertility Monitor (CBFM), which uses changes in urine levels of two key hormone metabolites for classifying the fertile window as “Low, High and Peak” readings,^2^ or line-based lateral flow assays for ovulation prediction.^3^ Several decades ago Blackwell and Brown^4^ developed the methodology for the first quantitative urine hormone devices, and now there are several (four) newer devices available such as the Mira monitor,^5^ Proov system,^6^ Inito Monitor,^7^ and Oova Monitor.^8^ While follow-up studies are underway for all of these monitors, our group has focused on validation studies using the Mira monitor with our previous pilot data showing user satisfaction and ease of use in participants with regular cycles.^5^

The Mira monitor and the CBFM are at-home testing systems that have been previously described in detail.^5,9^ They both use disposable test sticks to measure estrone-3-glucuronide (E_1_3G) and luteinizing hormone (LH) in the urine. The CBFM uses an optical intensity-based measurement, while Mira uses a fluorescence assay. Similar to the CBFM, the Mira’s LH test is the classical sandwich assay and the E_1_3G test is a competition assay. So as LH in the urine rises, the fluorescent intensity of the LH line rises, and for E_1_3G as its concentration rises, the corresponding fluorescent intensity of the E_1_3G line decreases.

Although the CBFM has been validated with both serum hormonal levels and ultrasound assessment of ovulation,^10,11^ Mira has yet to be validated to these external measures. We previously initiated validation studies of Mira in regular cycles by comparing it with the CBFM in a pilot study.^5^ A strong correlation between the CBFM estimated day of ovulation and the Mira LH surge (R = 0.98, *p* < 0.001) was found, and the changes in both E_1_3G and LH on the CBFM were reflected in the quantitative hormone changes found with Mira.

To date, preliminary studies have described the use of Mira in both the postpartum^12^ and perimenopause^13^ transitions, but without a comparison to another method of hormonal monitoring. In this current study, we seek to add to the validation of Mira by comparing it to the CBFM in the postpartum and perimenopause transitions in fertility, thereby extending our previous results in regularly cycling women.^5^

## Materials and Methods

### Design and recruitment

This was a retrospective study and included already collected menstrual cycle data from 16 North American women between the ages of 18 and 55 with user experience with both Mira and the CBFM during the postpartum or perimenopause periods. One participant had a collection over two postpartum periods from two different pregnancies (2 years apart). Participants were originally recruited as part of a larger dataset of women tracking their menstrual cycles with fertility monitors. Women were defined as postpartum if they had an infant within the last year. Perimenopause women were defined, based on Stages of Reproductive Aging Workshop (STRAW) criteria,^14^ as early perimenopause if they were age over 40 and had persistent 7-day or greater differences in cycle lengths and late perimenopause if they had >60-day intervals of amenorrhea. Participants were excluded if they were on medications that may impair or stimulate ovulation in the previous three months (hormonal contraceptives, ovulation induction medications, and hormone therapy), had known conditions impairing fertility (specifically: pelvic inflammatory disease, endometriosis, polycystic ovarian syndrome, or pituitary adenomas), had surgeries impacting the menstrual cycle (specifically: hysterectomy or bilateral oophorectomy; other surgeries were not exclusions), or if they were pregnant.

This study was approved by the health research ethics board at Marquette University (HR 4276, April 4, 2023). Participants provided consent to share their menstrual-charting data and medical history through Microsoft Forms including CBFM results, while their quantitative hormone data from Mira were acquired from an online Mira portal where users can share their data with their health care providers.

### Statistical analysis

**FIG. 1.**
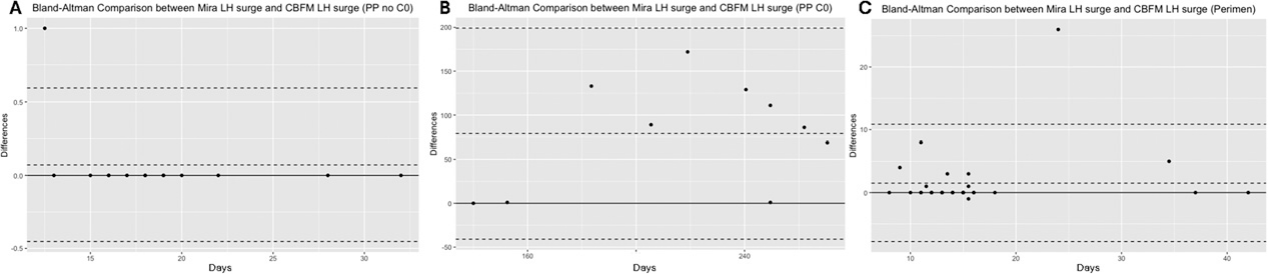
Bland–Altman comparison between CBFM and Mira LH surge days. The 0 line on the *x*-axis represents the mean of the two measures, and the *y*-axis represents the difference of each measure from that mean. **(A)** Postpartum Mira LH peak days are not significantly different from CBFM LH peak days in transition cycles after the first menses postpartum (t = 1.0, *p* = 0.34, “PP no C0”) but are significantly different prior to the first menses postpartum (**[B]**, t = 4.1, *p* = 0.003, “PP C0”) because of the long and variable pattern of amenorrhea before the first ovulation and menses. **(C)** Perimenopause Mira LH peak days are not significantly different from CBFM LH peak days (t = 1.8, *p* = 0.08); however, the limits of agreement are wider given the irregular cycles in perimenopause. CBFM, ClearBlue Fertility Monitor; LH, luteinizing hormone.

The primary analysis compared the LH hormone surge identified on the CBFM (first “Peak” day, day 0) to the LH surge on Mira (day of highest LH in the cycle, over a threshold of 11 mIU/mL based on the original pilot study)^5^ using Bland–Altman method agreement analysis.^15^ The Bland–Altman plot shown in Figure 1 is the same statistical method as the Tukey mean-difference plot and is used instead of Pearson’s correlation coefficients because it assesses the agreement between two assays measuring the same phenomenon (whereas Pearson’s correlation compares the correlation between two different phenomena). The 0 line on the *x*-axis represents the mean of the two measures, and the *y*-axis represents the difference of each measure from that mean.

In our secondary analyses, we compared the transition from low to high on the CBFM with the quantitative change in E_1_3G on Mira and compared the transition from high to peak on the CBFM to the quantitative change in LH on Mira using a univariate analysis of variance with a post-hoc Tukey test. Comparisons were between perimenopause, postpartum, and the original pilot (regular cycles)^5^ groups.

Demographic and charting data were compiled, analyzed, and visualized using R software (R version 4.3.2, The R Foundation for Statistical Computing) and SPSS (version 29, IBM). Mean and standard deviation were used for continuous variables (age, body mass index [BMI]), and median and interquartile intervals were used for discrete variables (pregnancies and miscarriages). Missing data were excluded pair-wise for each analysis. With 16 participants, we were powered to detect 1-day differences in the LH surge (80with correlation coefficients % power, with an alpha of 0.05, effect-size of 0.5, calculated with G*Power 3.1).

## Results

Demographics for the two groups are summarized in Table 1. An average of 3 cycles per participant in the postpartum group were collected (range 1–6), and an average of 4 cycles per participant in the perimenopause group were collected (range 1–9); see Table 2.

**Table 1.** Participant Characteristics (Mean, SD, Range for Continuous Data and Media, IQR for Discrete Data) in Postpartum and Perimenopause (*n* = 16)

		Postpartum *n* = 8 + 1^a^	Min	Max
Age	Mean, SD	32.3, 3.4	28.0	40.0
BMI	Mean, SD	22.5, 2.2	19.7	25.7
Pregnancies	Median, IQR	4, 4–5	2	8
Miscarriages	Median, IQR	0, 0–1.5	0	2

		Perimenopause *n* = 8	Min	Max
Age	Mean, SD	45.3, 3.2	40.0	51.0
BMI	Mean, SD	25.6, 4.7	20.1	33.6
Pregnancies	Median, IQR	6, 3.5	5	10
Miscarriages	Median, IQR	2, 1	0	4

^a^ One participant contributed two postpartum periods, so she is counted a second time with her older age and adjusted BMI from the pregnancy for the second postpartum period.

BMI, body mass index; IQR, interquartile interval; SD, standard deviation.

**Table 2.** Comparison of CBFM LH Surge Days to Mira LH Surge Between the Original Pilot Study^5^ and the Two Groups in the Current Study

CBFM day relative to peak	Postpartum *n* = 8 + 1 cycles = 24	Perimenopause *n* = 8 cycles = 33	Original pilot,^5^ *n* = 21 cycles = 57
	Frequency of Mira peak	Frequency of Mira peak	Frequency of Mira peak
CBFM 1 day before Peak	3 (13%)	1 (3%)	5 (9%)
CBFM first Peak	14 (58%)	24 (73%)	37 (65%)
CBFM second Peak	0%	2 (6%)	12 (21%)
Outliers	7 (29%)	6 (18%)	3 (5%)
Total ±1 day of P	71%	82%	95%

CBFM, ClearBlue fertility monitor; LH, luteinizing hormone.

Bland–Altman analysis demonstrated good agreement between the CBFM and Mira LH surges (i.e., no significant difference) in the postpartum transition cycles after the first postpartum menses (t = 1.0, *p* = 0.34, Fig. 1A) and the perimenopause group (t = 1.8, *p* = 0.08, Fig. 1C) but poor agreement between the CBFM and Mira for postpartum amenorrhea before the first menses (t = 4.1, *p* = 0.003, Fig. 1B).

In the original pilot^5^ with regular cycles, most (95%) of the Mira LH surges fell within ±1 day of the CBFM LH surge. However, in the postpartum group in this study, only 71% of Mira LH surge fell within ±1 day of the CBFM LH surge, and in the perimenopause group, 82% fell within ±1 day of the CBFM LH surge (Table 2).

**FIG. 2.**
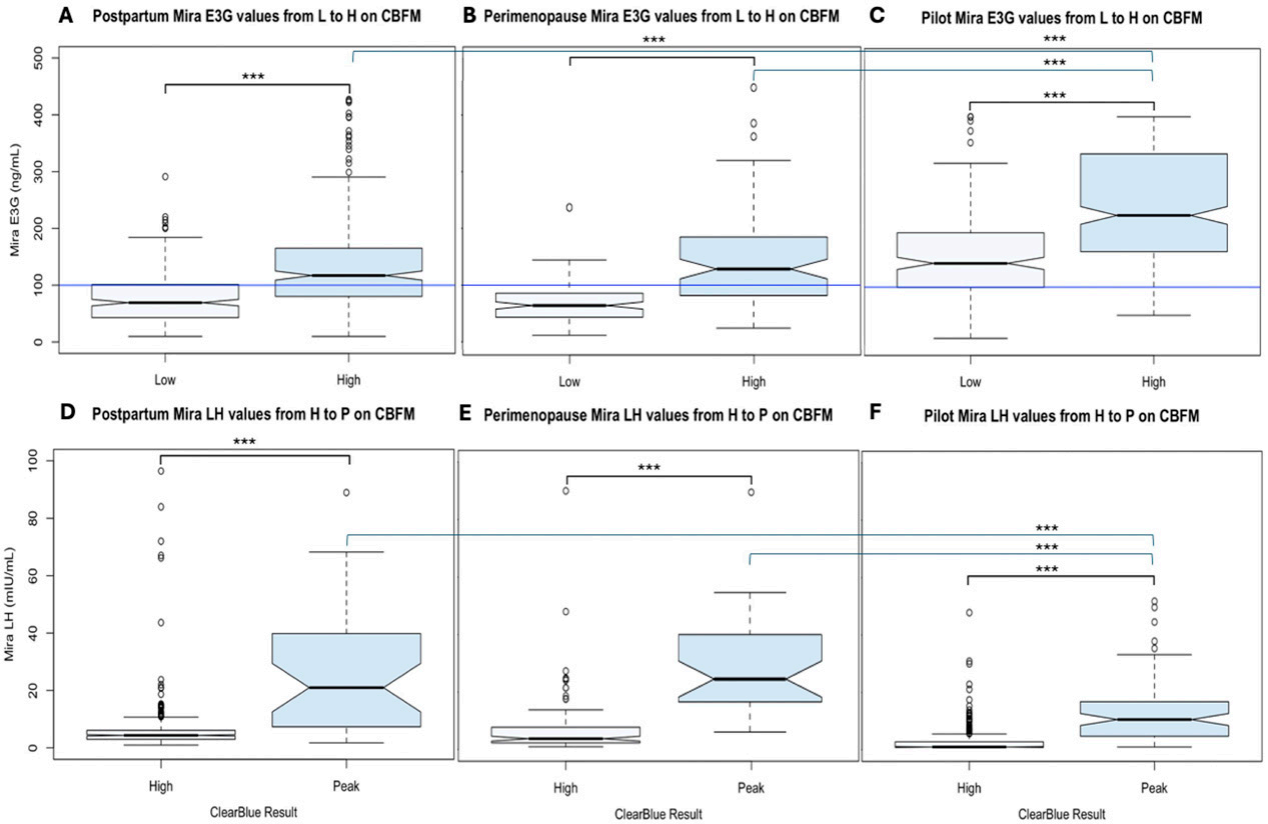
**(A–C)** Mira E_1_3G values (ng/mL) relative to the CBFM Low (L) to high (H) transition. The rise in Mira E_1_3G from L to H on CBFM was significantly different (***p < 0.001) in all three cases: pilot **(A)**, postpartum **(B),** and perimenopause **(C)**. Mira E_1_3G values were higher overall on L and H days in the pilot than in both postpartum and perimenopause. **(D–F)** Mira LH values (mIU/mL) relative to the CBFM high to peak (P) transition. The rise in Mira LH from L to H on the CBFM was significantly different in the pilot **(D)**, postpartum **(E)**, and perimenopause **(F)**. On the CBFM peak day, Mira LH values were higher overall in the postpartum **(E)** and perimenopause **(F)** than in the pilot **(D)**. E_1_3G, estrone-3-glucuronide.

Figure 2 shows the Mira E_1_3G values between a “Low” and “High” reading on the CBFM, for the postpartum cycles (Fig. 2A), perimenopause cycles (Fig. 2B), and the pilot study of regular cycles (Fig. 2C). In all three groups, the Mira E13G levels were significantly higher for the CBFM “High” compared with “Low” (all *p* < 0.001). The mean difference in Mira E_1_3G levels from “Low” to “High” was 59.5 (±9.1) for the postpartum cycles, 63.3 (±7.1) for perimenopause cycles, and 97.5 (±9.4) for pilot study regular cycles. Similarly, Figure 2 shows the Mira LH values between a “High” and “Peak” reading on the CBFM, for postpartum cycles (Fig. 2D), perimenopause cycles (Fig. 2E), and the pilot study of regular cycles (Fig. 2F). In all three groups, the Mira LH levels were significantly higher for the CBFM “Peak” compared with “High” (all *p* < 0.001). The mean difference in Mira LH levels from “High” to “Peak” was 22.5 (±1.5) for the postpartum cycles, 23.1 (±1.4) for perimenopause cycles, and 9.8 (±1.1) for the pilot study regular cycles. Tukey post-hoc comparisons were all significant for the above comparisons (*p* < 0.001).

Of interest, E_1_3G was significantly higher overall (*p* < 0.001) on both CBFM “Low” and “High” days in the regularly cycling women in the pilot (Fig. 2C) compared with both the postpartum (Fig. 2A) and perimenopause groups (Fig. 2B), but E_1_3G levels were not significantly different between the postpartum and perimenopause groups. In contrast, LH was significantly lower overall on CBFM “Peak” days in the regular cycling group in the pilot (Fig. 2F) compared with the postpartum and perimenopause groups (*p* < 0.001). The LH levels were not significantly different between the postpartum and perimenopause groups (Fig. 2D, E).

**FIG. 3.**
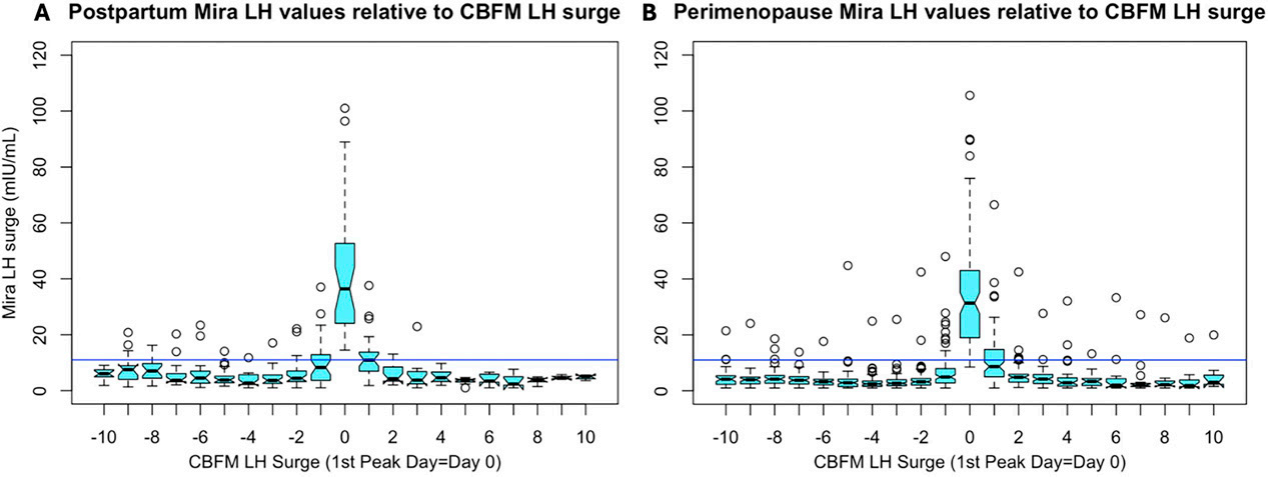
LH values leading up to and after ovulation in the postpartum **(A)** and perimenopause **(B)** groups. Mira LH values (in mIU/mL) relative to the CBFM LH surge (1st CBFM peak = day 0). **(A)** All postpartum participants. **(B)** All perimenopause participants. The Mira LH surge is highly concentrated around the CBFM LH surge (day 0), with some signal on day −1 and day +1. Open circles outside of these days above the blue threshold line (11 mIU/mL) represent aberrant LH surges outside the CBFM LH surge.

The Mira LH changes leading up to the CBFM LH surge in both the postpartum and perimenopause groups show a sharp rise on day 0 (Fig. 3A, B). A threshold line of 11 mIU/mL (blue line, Fig. 3) has been suggested in other studies (5 – correct to reference 5) as a potential threshold to trigger ovulation and is added here as a reference. There were outliers, or aberrant LH surges on the Mira monitor (open circles, Fig. 3) identified in both groups with LH levels >11 mIU/mL that did not coincide with the CBFM peak day (day 0).

**FIG. 4.**
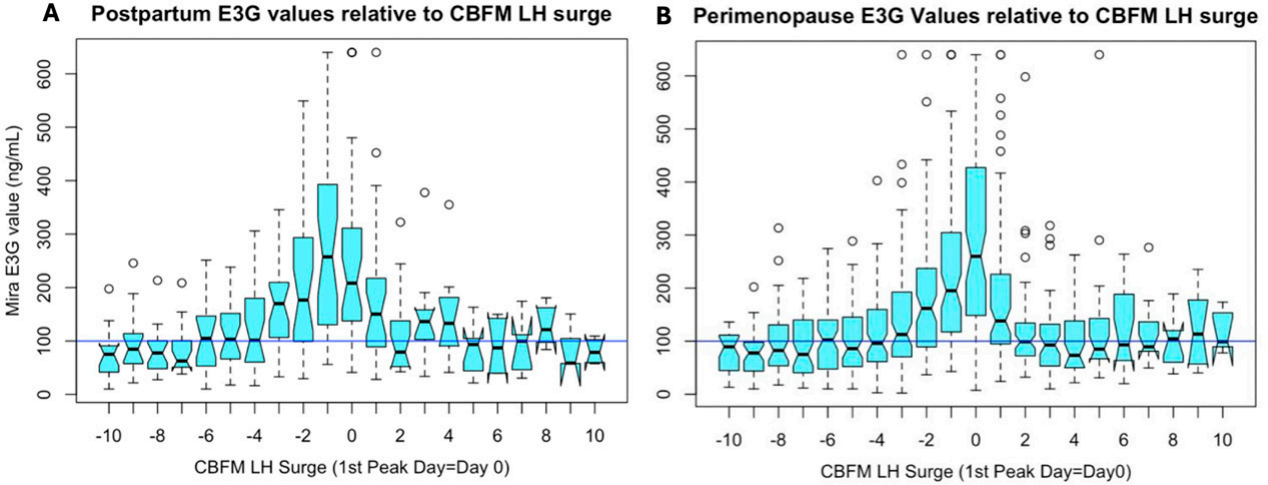
E_1_3G values leading up to and after the CBFM LH surge in the postpartum **(A)** and perimenopause **(B)** groups. **(A)** Postpartum E_1_3G values relative to the CBFM LH surge (1^st^ “Peak” Day = day 0). **(B)** Perimenopause E_1_3G values relative to the CBFM LH surge. The threshold of E_1_3G > 100 (blue line) has been proposed as a threshold for the fertile window in other studies.^12,13^

The Mira E_1_3G changes leading up to the CBFM LH surge in both postpartum and perimenopause groups showed a gradual rise (Fig. 4), with highest median E_1_3G levels on the day of the CBFM LH surge (day “0”) in the postpartum group (Fig. 4A) and day 0 in the perimenopause group (Fig. 4B). A threshold of E_1_3G > 100 has been proposed in other studies^12,13^ and is shown in Figure 4 (blue line) as a reference.

## Discussion

We found that quantitative measurements of E_1_3G and LH in the urine as assessed by the Mira monitor showed good agreement with the CBFM in perimenopause and postpartum transition cycles but poor agreement during postpartum amenorrhea. This study extends our pilot results^5^ that showed that the CBFM and Mira monitor LH surges were highly correlated in normal cycles and support the validity of the Mira quantitative fertility monitor for monitoring of urine hormonal patterns for the menstrual cycle, with the caveat of poor agreement during postpartum amenorrhea.

One of the advantages of using a quantitative hormone monitor is that more dynamic hormone changes can be identified rather than threshold changes on traditional qualitative tests. This is especially helpful in the postpartum and perimenopausal periods when there can be transient rises in estrogen without leading to ovulation.^12,13^ There were multiple LH surges (non-ovulatory peaks) on both the CBFM and Mira during postpartum amenorrhea. The LH surge in the first ovulation postpartum may have to be higher to trigger ovulation given hypothalamic suppression postpartum. During the perimenopause, aberrant LH patterns were found with far more LH surges that appear to be non-ovulatory (Fig. 2) than were found in the pilot study in regular cycles.^5^ This may be related to the ovaries having lower sensitivity to LH, akin to the postpartum amenorrheic period, but in this case due to lower ovarian egg reserve and potential impaired follicular development.^16,17^ It may be that gonadotropin-releasing hormone pulse frequency may lead to higher LH levels, as has been shown in the case of polycystic ovarian syndrome, which also leads to abnormal LH patterns.^18^

Postpartum and perimenopause E_1_3G levels were significantly lower than in women with regular cycles (Fig. 4A, B). Lower E_1_3G levels were present in women during postpartum amenorrhea who have an ovarian quiescent pattern without follicular activity as we have previously shown.^12,19^ Further observation in the transition to regular cycles postpartum may be able to show a shift in E_1_3G levels in the follicular phase as follicle development returns to normal.^20^ It is also not surprising that there are changes in E_1_3G levels in the perimenopause, and some of the changes in E_1_3G in the luteal phase (Fig. 3B) may reflect luteal out-of-phase follicular events that can be observed in the menopausal transition.^16^

A limitation of this study was the small sample size; however, it was adequately powered to identify a significant correlation with the LH surge between the CBFM and Mira monitors. Another limitation is that Mira was not directly compared with ultrasound-confirmed ovulation, with the previously ultrasound-validated CBFM used as a proxy.^21^ Strengths of the study include the daily measurements of urine hormonal levels over weeks, which provides more information on hormonal patterns than intermittent serum measurements. In addition, these monitors provide measurements of not just the LH surge (as in industry standard tests for ovulation prediction with qualitative line-based tests)^22^ but also E_1_3G as a metabolite of serum estrogen.

The next step for validating the Mira monitor will be to correlate the urine hormone results to the gold standard of ultrasound-confirmed ovulation, as well as to serum hormone concentrations, which was previously completed for the CBFM^10,11,23^ and for Quidel-based urinary assays.^24^ This further validation study to correlate the Mira monitor to ultrasound ovulation is currently in progress.^9^ Once validated, a potential future application of quantitative urine hormonal monitoring is for the prediction of the timing of menopause. Attention has been given to perimenopause menstrual cycle variation in the STRAW + 10 criteria, which provides a model for predicting when menopause is likely to occur.^14^ There are other models that have included self-reported symptoms and lab correlates in menopause prediction models.^25^ Other studies have suggested that variability in estrogen secretion leading up to menopause may cause uncoordinated follicular development.^16^ Thus, daily hormonal monitoring of E_1_3G could possibly contribute to these predictive models for menopause.

## Conclusions

Quantitative urine hormone monitoring of E_1_3G and LH with the Mira monitor was highly correlated to qualitative readings on the ultrasound-validated CBFM. While direct comparison of the Mira monitor to ultrasound-confirmed ovulation is necessary, it is possible that urine quantitative hormonal assessments could be helpful for clinical applications in the future, specifically for tracking menstrual cycle irregularity and hormone variability in postpartum and perimenopause women.

## Author’s Contibution

T.P.B. (Conceptualization, Data curation, Formal analysis, Investigation, Methodology, Project administration, Resources, Software, Supervision, Validation, Visualization, Writing—original draft, review & editing). P.K.D.-B. (Formal analysis, Methodology, Supervision, Validation, Writing—original draft, review & editing). P.J.Y. (Conceptualization, Formal Analysis, Investigation, Methodology, Supervision, Validation, Writing—original draft, review & editing). R.F. (Conceptualization, Formal analysis, Methodology, Supervision, Validation, Writing—original draft, review & editing). M.S. (Conceptualization, Data curation, Formal analysis, Investigation, Methodology, Project administration, Resources, Software, Supervision, Validation, Writing—original draft, review & editing).

## Abbreviations Used

CBFM: ClearBlue Fertility Monitor
E13G: estrone-3-glucuronide
LH: luteinizing hormone
STRAW: Stages of Reproductive Aging Workshop
